# Tethered UAV Autonomous Knotting on Environmental Structures for Transport

**DOI:** 10.34133/cbsystems.0450

**Published:** 2025-12-26

**Authors:** Rui Jin, Xinhang Xu, Yizhuo Yang, Jianping Li, Muqing Cao, Lihua Xie

**Affiliations:** ^1^ School of Electrical and Electronic Engineering, Nanyang Technological University, Nanyang 639798, Singapore.; ^2^ Robotics Institute, Carnegie Mellon University, Pittsburgh, PA 15213, USA.

## Abstract

Cable-driven robotic systems are widely adopted for transport tasks due to their high load-bearing efficiency. However, their deployment in unstructured or unknown environments is hindered by the challenge of rapidly and reliably anchoring the cable endpoint. This work introduces a deployable cable-driven transport system that combines a tethered unmanned aerial vehicle (UAV) with a winch mechanism to autonomously form a topologically stable entanglement for cable anchoring. At the core of the system is a modular knot planner that integrates human-in-the-loop enclosing plane extraction, frontier-based enclosing path search, and knotting trajectory generation, incorporating metrics such as enclosing planarity, tether visibility, and tether clearance. In real-world experiments conducted in an urbanized outdoor environment, the system autonomously interpreted high-level user commands, executed a full knotting operation around a target structure, and successfully lifted a 15.3-kg payload to a height of 3.5 m. Beyond real-world trials, simulation studies confirmed the system’s shape-agnostic knotting capability. A set of ablation experiments further demonstrated the necessity and effectiveness of these joint optimization metrics. Together, these results highlight the practicality and robustness of the proposed system for autonomous heavy-load transport in complex and previously unprepared environments, offering new capabilities for rapidly deployable robotic logistics.

## Introduction

Cable-driven robotic systems have been widely adopted for object transport in diverse scenarios, including space exploration [1], logistics [2], and biological study [3]. These systems offer 2 primary advantages. First, the cable’s ability to transmit forces over long distances enables such systems to operate effectively in large and distributed workspaces. Second, directly coupling the motor to the cable allows efficient torque-to-tension conversion with minimal energy loss, giving these systems a higher load-carrying capacity than wheeled [4], legged [5], or aerial robots [6–9].

However, cable-driven systems can only apply force along the cable direction and the cable’s endpoint must be anchored before performing tasks, restricting their use to predesigned environments. To extend their applicability, enabling them to perform transport tasks in unstructured settings remains an open problem. Addressing this issue involves tackling several practical challenges, analyzed as follows:

The first challenge lies in flexible anchoring. Each transport task requires the cable to exert force in a specific direction, which demands spatial diversity of anchor points. Moreover, given the uncertainty of the environment, the system needs the capability to anchor the cable onto structures with a wide range of geometric configurations. These requirements collectively demand flexibility, requiring the system to possess sufficient mobility to position the cable endpoint at diverse locations.

The second challenge lies in reliable anchor point selection. It is nontrivial to determine how to choose a point that enables easy and effective anchoring, avoids excessively cluttered surroundings or infeasible geometries, and is located on structures with sufficient strength to support heavy payloads. These factors collectively define reliability, requiring the selection module to evaluate both structural feasibility and mechanical stability.

The third challenge lies in autonomous anchoring. Anchoring the cable at spatially diverse locations, particularly elevated positions, can render manual intervention infeasible due to limited human accessibility. In addition, performing anchoring actions with high placement precision in unknown environments via teleoperation imposes significant demands on human operators. These factors collectively increase the need for autonomy, requiring the system to perform anchoring autonomously even in geometrically complex and partially known environments.

**Fig. 1.**
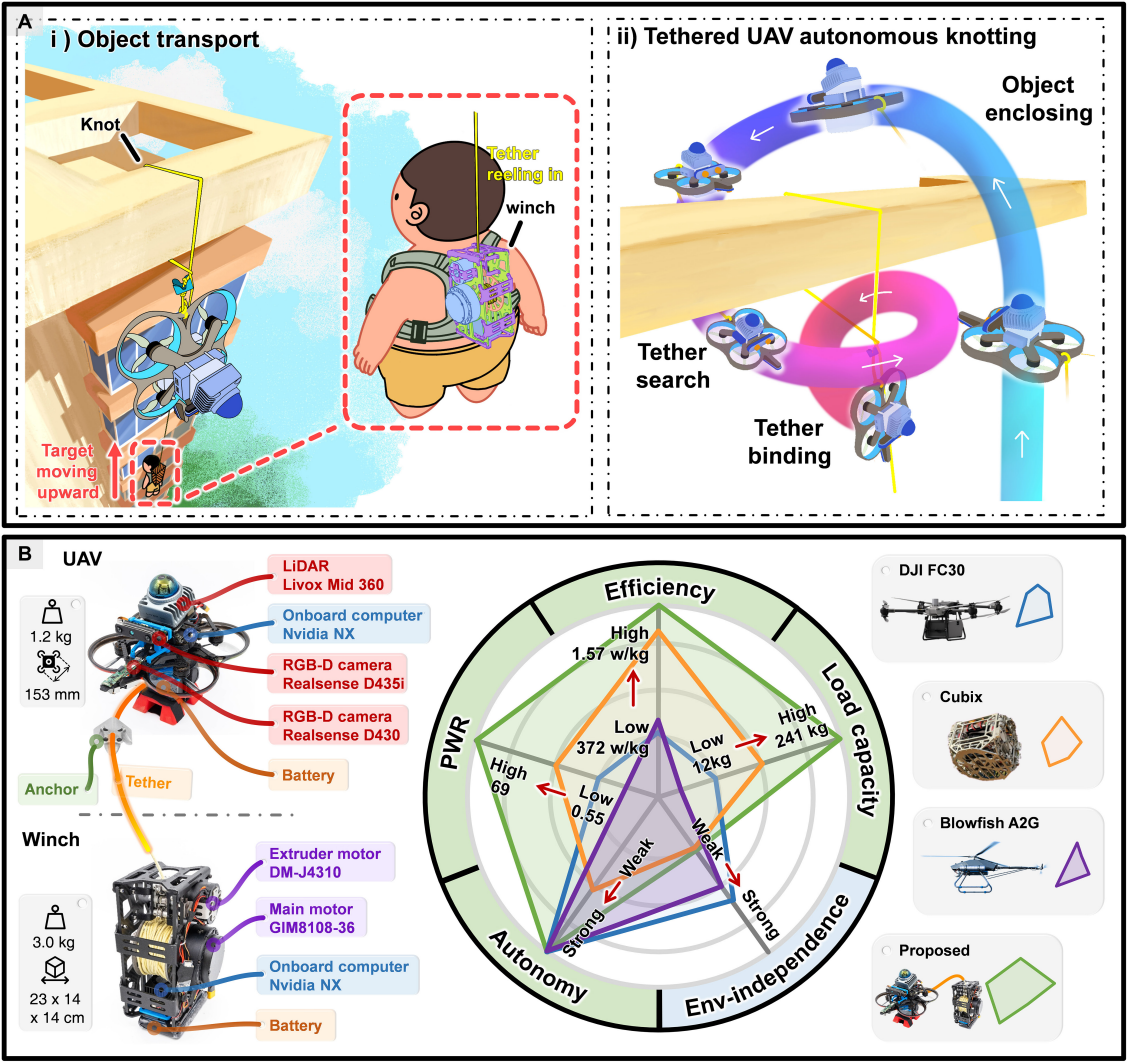
Overview of the proposed autonomous transport system. (A) Object transport demonstration. (i) The full process of lifting the target object. After the tethered UAV autonomously performs knotting on top of a tall building, the winch reels in the tether to lift the object. (ii) Autonomous knotting procedure. The UAV first performs object enclosing, then enters the tether search state, and finally executes tether binding to complete knotting. (B) Radar chart comparing the proposed system with other state-of-the-art aerial/cable-driven transport robots, including the commercial high-payload multicopter (DJI FC 30), commercial high-payload helicopter (Blowfish A2G), and a portable wire-driven parallel robot (Cubix). PWR (payload-to-weight ratio) is the metric describing the ratio of the maximum payload capacity to the weight of the robot. The outer ring of the radar chart is color-matched to the best-performing system for each corresponding metric. For commercial platforms, we obtained the necessary data from the official manufacturer’s websites [36,37], and for Cubix, the data are extracted from Refs. [14, 30].

Based on the above analysis, we propose a complete cable-driven transport system that features autonomous knotting by a tethered unmanned aerial vehicle (UAV) to anchor the cable endpoint onto environmental structures (Fig. 1). (a) To enhance flexibility, the system employs a tethered UAV as the mobile platform and adopts knotting as the anchoring method. The UAV’s agility and mobility [10] enable it to carry the cable endpoint and freely navigate to fly around the surface of the target object and form a closed loop. Moreover, the use of knotting allows anchoring operations to adapt to various environmental structures with a wide range of unstructured geometries; (b) To improve the reliability of anchor point selection, we incorporate human prior knowledge into the process by leveraging user-drawn sketches on captured RGB images. These sketches serve as intuitive cues to guide the system in identifying semantically meaningful and physically feasible anchoring locations, thereby combining human semantic understanding with the UAV’s precise execution in a complementary manner; (c) To enhance the autonomy of knotting in complex environments, we develop a comprehensive planning framework, knot planner, which is guided by user input to generate safe and executable trajectories that enclose the target structure and bind the tether. The knot planner consists of 3 key modules, including the human-in-the-loop enclosing plane extraction (HEPE), frontier-based enclosing path search (FEPS), and knotting trajectory generation (KTG). As the front-end modules of the planner, HEPE extracts the desired enclosing plane from the user sketch to guide the overall knotting process, and FEPS detects frontier clusters on the surface of the target structure and searches for enclosing paths that tightly wrap around it.

In the back end, KTG initializes the optimization using the path generated by FEPS and produces the final trajectory for UAV execution. The optimization considers both general motion metrics and task-specific terms designed for knotting, including the enclosing planarity (EP), tether visibility (TV), and tether clearance (TC). In summary, the main contributions of this work are as follows:

• We propose a complete tethered UAV-based system capable of performing autonomous knotting on unseen environmental structures for transport.
• We propose knot planner, which generates safe and executable trajectories for UAVs to accomplish knotting tasks, guided by user signals.
• We conduct extensive experiments in both simulation and real-world environments to validate the effectiveness and robustness of the proposed system.

### Related work

Anchoring a tether to environmental structures is a critical capability for a cable-driven transport system. Existing approaches primarily utilize either mechanical fixtures [11–13] or topological configurations [14–18] (e.g., knotting on objects). However, mechanical fixtures often lack generality and struggle to adapt to arbitrary attachment points.

By contrast, topological methods are mainly realized through different forms of knotting or entanglement, thereby offering greater flexibility for diverse structures. One method employs variable-size slip knots, in which robotic end-effectors place a tether loop around the target and tighten it to achieve fixation [15]. A more fundamental method is winding, where the tether is wrapped around a structure to generate a frictional holding force [16]. To improve the precision and robustness of winding-based anchoring, payload-assisted anchoring with a suspended pod has been proposed [17], where the pod cooperates with the drone to execute wrapping loops and assist disentangling, thereby enhancing stability and energy efficiency. Furthermore, to reduce the number of required winding loops and ensure stability under heavy loads, specialized anchors such as hooks or latching devices have been introduced [14,18]; these lock the topological configuration after a single wrapping by sliding along the tether and securing the topological structure when tensioned. Despite these advances, most topological methods still require prior geometric knowledge of target structures or predefined markers to close the loop around the object. They typically require prior geometric knowledge of specific wrapping targets or rely on predefined environmental markers to form a closed loop around the target object. To address these limitations, a more general and adaptable anchoring method is required, which can perceive unknown geometry.

In this context, using tethered UAVs to finish autonomous topologically based anchoring exhibits significant parallels with 3-dimensional (3D) exploration and full coverage [19–25]. This is because successful knotting necessitates that the UAV actively explores the space, precisely perceives key target objects (e.g., estimating the size, position, and orientation of pillars or beams), and plans flight trajectories, ensuring that the tether effectively encircles or passes through these targets.

While existing trajectory planning methods for tasks such as exploration and full coverage share certain similarities with these requirements, they are fundamentally ill-suited for autonomous knotting in environments with uncertainty. In methods designed for common UAVs [19–25], the dynamic state of the tether and its interaction with the environment are typically neglected, placing the system at risk of negative consequences such as entanglement with obstacles. Such entanglement can severely compromise maneuverability. On the other hand, algorithms developed specifically for tethered UAVs [26–29] are concerned with generating entanglement-free paths, but they lack the capability to autonomously form robust topological configurations, such as the knots required for secure anchoring in complex and unstructured environments. To the best of our knowledge, no existing algorithm is capable of performing autonomous knotting in unknown environments.

## Materials and Methods

### System architecture

As shown in Fig. 1A, the knotting process consists of 3 stages: object enclosing, tether search, and tether binding. Specifically, similar to the approach in Ref. [30], during object enclosing and tether search, the UAV carries the endpoint of the tether, navigates around the surface of the target object, and locates the suspended tether. In tether binding, the UAV circles around the tether and tightens it using its own gravity together with the winch, allowing the anchor to achieve entanglement for knotting. Fig. 1B illustrates the hardware architecture, where the UAV is tethered to the winch via a cable and equipped with an onboard IMU, LiDAR, and RGB-D cameras, enabling it to perceive both the target structure, the tether, and the surrounding environment. With the onboard computer, the UAV can then autonomously perform an encirclement to form a closed loop around the object.

**Fig. 2.**
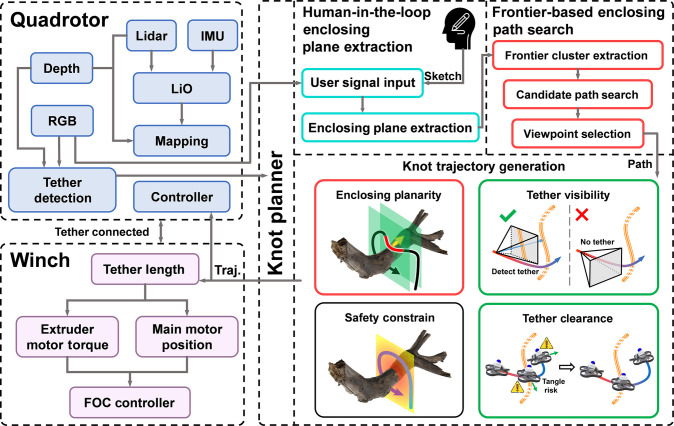
Module relationships and workflow of proposed system. The knot planner uses user signals to extract an enclosing plane, generates enclosing paths, and generates the final trajectory with general and knotting-specific metrics.

The winch, located at the far end relative to the UAV and connected to the transported object, is responsible for tether extrusion and retraction. In parallel, an anchor affixed to the tether at a designated distance behind the UAV serves to ensure that the knot is fastened with sufficient stability and reliability. Fig. 2 illustrates the software architecture: The UAV employs FAST-LIO [31] to estimate its odometry, constructs a voxel map by fusing point clouds from both the depth camera and the LiDAR, and, within this 3D discrete space, computes the distance from each voxel to the nearest obstacle to obtain the signed distance field (SDF). RGB and depth images are further used jointly to detect the tether. The winch system enables both tether length control and high load-bearing capacity by releasing the tether during knotting to allow UAV maneuvering, and reeling it in to perform high-load transport. To further endow the UAV&winch setup with autonomous knotting capability, we propose knot planner. To initiate the planning pipeline, the user can sketch on the captured RGB image to specify a desired enclosing region based on the HEPE module (Human-in-the-loop enclosing plane extraction section), which then guides the FEPS module (Frontier-based enclosing path search section) to find a path enclosing the target structure. The KTG module (Knotting trajectory generation section) further refines this path to produce final trajectories guided by knotting-specific metrics.

### Human-in-the-loop enclosing plane extraction

This module allows the user to provide minimal yet high-level guidance by sketching the target object on an RGB image captured by the UAV. Based on this input, the system infers a 3D enclosing region around the object, which is later used to constrain the subsequent FEPS and KTG modules.

#### User signal input

The user first draws a 2D line sketch on the RGB image streamed from the UAV, which is then projected onto the corresponding 3D line $\ell_{3D}$ defined in world coordinates.

**Fig. 3.**
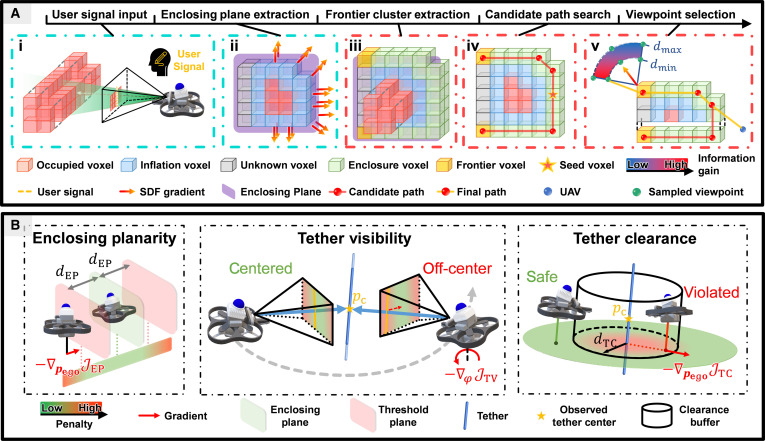
An illustration of the proposed knot planner. (A) Workflow of the object enclosure state. The user is prompted to draw a line on the front-view camera image to specify the desired knotting region. The enclosure plane is then determined based on both the user input and the signed distance field (SDF) of the observed knotting target. The knot planner iteratively selects candidate viewpoints to eliminate surface frontiers near this plane. (B) Visualization of the physical interpretation of the metrics.

Subsequently, as shown in Fig. 3A-i, a raycasting operation is performed from the camera position $p_{\mathrm{cam}}$ toward the midpoint of $\ell_{3D}$, with the first free voxel adjacent to an inflated occupied voxel $v_{\text{seed},\inf}$ along the ray selected as the seed voxel $v_{\text{seed}}$, as shown in Fig. 3A-i. A connected component region growing operation is then performed from $v_{\text{seed},\inf}$, constrained within the set of inflated occupied voxels. This process yields a voxel set $O_{\inf}$, representing the inflated volume of the estimated target object, and is formally defined as:

$$O_{\inf}=\mathrm{CC}\left(v_{\text{seed},\inf},V_{\inf\_\mathrm{occ}}\right)$$ (1)

where $\mathrm{CC}\left(\cdot \right)$ denotes the connected component region growing operation and $V_{\inf\_\mathrm{occ}}$ is the set of inflated occupied voxels.

#### Enclosing plane extraction

To prevent tether slippage during enclosure, the UAV is encouraged to fly within the plane approximately perpendicular to the object surface. We therefore utilize the observed surface geometry of the object to compute the enclosing plane $\Pi_{\mathrm{enc}}$. As shown in Fig. 3A-ii, to calculate $\Pi_{\mathrm{enc}}$, we first construct an intermediate sketch plane using $p_{\mathrm{cam}}$ and $\ell_{3D}$, and extract a subset of free voxels within a fixed distance threshold $d_{\text{sketch}}$ from this plane and in proximity to $O_{\inf}$, denoted as $O_{\text{sketch}}$, where $O_{\inf}$ is defined in Eq. (1). Next, we compute the SDF gradients for all voxels in $O_{\text{sketch}}$ using trilinear interpolation. These gradients are used to construct a covariance matrix $C_{\mathrm{SDF}}$, from which the eigenvector corresponding to the smallest eigenvalue is extracted as the estimated normal vector n of $\Pi_{\mathrm{enc}}$, as given by:

$$\begin{array}{c} n=\text{arg}\;\underset{e_{i}}{\text{min}}\;\lambda_{i},\;\text{where }C_{\mathrm{SDF}}e_{i}=\lambda_{i}e_{i} \end{array}$$ (2)

The normal vector n defines the orientation of the plane $\Pi_{\mathrm{enc}}$, which is anchored at the mean position p of voxels in $O_{\text{sketch}}$. The plane $\Pi_{\mathrm{enc}}$ is thus fully defined by the pair $\left(p,n\right)$.

### Frontier-based enclosing path search

The goal of this module is to generate front-end paths that wrap around the target object while remaining within a spatial metric defined by a fixed distance to the enclosing plane $\Pi_{\mathrm{enc}}$. The generated paths serve as guidance paths for the subsequent trajectory optimization module to generate the final knotting trajectory to be executed by the UAV.

#### Frontier cluster extraction

As shown in Fig. 3A-ii, an inflation operation is first performed on $O_{\inf}$, retaining only the free voxels whose center coordinates lie within a fixed distance to the plane $\Pi_{\mathrm{enc}}$. The resulting set is defined as the enclosing voxels $O_{\mathrm{enc}}$, formally given by

$$\begin{array}{c} O_{\mathrm{enc}}=\left\{v\in {\text{Inflate}}_{26}\left(O_{\inf}\right)\backslash O_{\inf}\;|\;\text{dist}\left(c\left(v\right),\Pi_{\mathrm{enc}}\right)\leq d_{\mathrm{enc}}\right\} \end{array}$$ (3)

where $c\left(v\right)$ denotes the center coordinate of voxel v, $d_{\mathrm{enc}}$ is the distance threshold to the enclosing plane, and ${\text{Inflate}}_{26}\left(\cdot \right)$ represents the 26-connectivity dilation.

Next, we extract frontier voxels from $O_{\mathrm{enc}}$ by identifying those that are adjacent to unknown map regions. These frontier voxels are then grouped into frontier clusters based on spatial connectivity. For each cluster, we compute the mean voxel $v_{f\;,i}$, representing the cluster center, as well as the average SDF gradient direction $g_{f\;,i}$, which indicates the local surface normal or boundary transition direction.

#### Candidate path search

To generate a path that satisfies the topological requirement of target object enclosing, we first compute a candidate path. This path is then connected to the UAV’s current position and the selected viewpoint (as discussed in the Frontier-based enclosing path search section) to form the final enclosing path.

In Fig. 3A-iii, we select 2 distinct frontier clusters and use the A* algorithm [32] to search 2 paths from the seed voxel $v_{\text{seed}}$ to the mean voxel of each cluster, constrained within the enclosing voxel set $O_{\mathrm{enc}}$.

These 2 paths are then concatenated head-to-tail into a single candidate path, denoted as $P_{\text{cand}}$, whose direction is aligned with a predefined winding orientation around the target object.

#### Viewpoint selection

To guide the UAV to enclose the target object, the viewpoint with the highest expected information gain is selected as the navigation target (Fig. 3A-iv). Based on a predefined enclosing orientation around the object, the frontier cluster containing the endpoint of the candidate path is identified as the expected eliminated region and indexed as e. Then, a set of candidate viewpoints are generated by uniformly sampling positions over an annular sector centered at the cluster’s mean voxel $v_{f\;,e}$. The sector is defined by an inner radius $d_{\min}$, outer radius $d_{\max}$, and angular span θ. It lies in a plane parallel to $\Pi_{\mathrm{enc}}$, and its central axis is aligned with the projection of the average SDF gradient direction ${\bar{g}}_{f\;,e}$ onto $\Pi_{\mathrm{enc}}$.

Each candidate viewpoint is defined as $vp_{j}=\left(p_{j},n_{j}\right)$, where $p_{j}$ is the 3D position and $n_{j}\in \left\{-1,\;1\right\}$ indicates the UAV’s heading direction within the sector plane. To evaluate the utility of a viewpoint for frontier elimination, we define the information gain of $vp_{j}$ based on the set U of unknown voxels that (a) are visible from $vp_{j}$; (b) are connected to the inflated object volume $O_{\inf}$ through unknown voxels; and (c) belong to the enclosing region $O_{\mathrm{enc}}$.

To favor observations near the target frontier, we assign each visible voxel $u\in U(vp_{j})$ a distance-based weight relative to the viewpoint position $p_{j}$. The information gain of viewpoint $vp_{j}$ is computed as:

$$\mathrm{IG}\left(vp_{j}\right)=\sum_{u\in U}\text{exp}\left(-\alpha \left\|u-p_{j}\right\|\right)$$ (4)

where α>0 is a decay coefficient. Finally, the candidate with the highest gain is selected:

$$vp^{*}=\text{arg}\underset{vp_{j}}{\text{max}}\mathrm{IG}\left(vp_{j}\right)$$ (5)

To finalize the enclosing path, the current UAV position $p_{\text{odom}}$ is prepended to $P_{\text{cand}}$ as the initial point. The resulting path is then simplified using ray validity checks, yielding the final enclosing trajectory $P_{\mathrm{enc}}$.

Once the target frontier has been eliminated through the enclosing maneuver, we construct a return path to bind the wire. To avoid retracing the same region, a separating plane is used to exclude the previously traversed space, which is marked as invalid, and an A* search is performed to compute the return path.

### Knotting trajectory generation

As discussed in Introduction, autonomy is critical for the knot planner. To support autonomous execution, we introduce a series of task-oriented metrics that guide the generation of feasible and effective trajectories for the knotting task. These include metrics on satisfying the user-specified EP, maintaining the TV within the UAV’s field of view (FoV), and preserving a safe TC between the UAV and the tether during the final knotting motion. In the following, we denote the UAV’s position and yaw angle as $p_{\mathrm{ego}}\in {\mathbb{R}}^{3}$ and $\psi \in \mathrm{SO}\left(2\right)$, respectively.

#### Enclosing planarity

The first metric EP requires that the UAV trajectory during the object enclosing state remains within a fixed distance from the enclosing plane $\Pi_{\mathrm{enc}}$, which is approximately perpendicular to the object surface, to prevent tether slippage.

The penalty is determined by the signed distance from the UAV position $p_{\mathrm{ego}}$ to the plane $\Pi_{\mathrm{enc}}$, computed as

$$s=n^{⊤}p_{\mathrm{ego}}-d$$ (6)

where n is the normal vector of $\Pi_{\mathrm{enc}}$ and d∈ℝ is its signed distance to the origin.

Based on this signed distance, the corresponding penalty is defined by the cost function

$$J_{\mathrm{EP}}\left(p_{\mathrm{ego}}\right)=\frac{1}{2}\cdot {\left(\text{max}\left(0,|s|-d_{\mathrm{enc}}\right)\right)}^{2}$$ (7)

where $d_{\mathrm{enc}}>0$ specifies the allowable deviation from the plane and is consistent with the threshold used to define the enclosing voxels. This cost remains zero when $p_{\mathrm{ego}}$ lies within the acceptable range and increases quadratically once the deviation exceeds the threshold.

The gradient of the cost with respect to $p_{\mathrm{ego}}$ is

$$\frac{\partial J_{\mathrm{EP}}}{\partial p_{\mathrm{ego}}}=\text{max}\left(0,|s|-d_{\mathrm{enc}}\right)\cdot \text{sign}\left(s\right)\cdot n$$ (8)

which guides the trajectory back toward the valid region only when a violation occurs.

#### Tether visibility

To encourage the UAV to maintain visual alignment with the tether during the tether binding state, we introduce a yaw alignment cost based on the discrepancy between the current yaw ψ and the expected yaw $\psi_{e}$, which points from the UAV to the observed tether center $p_{c}$. The expected yaw is computed as:

$$\begin{array}{c} \psi_{e}\left(p_{\mathrm{ego}},p_{c}\right)=\text{a}\text{tan}2\left({\left(p_{c}-p_{\mathrm{ego}}\right)}_{y},\;{\left(p_{c}-p_{\mathrm{ego}}\right)}_{x}\right) \end{array}$$ (9)

and the corresponding alignment cost is defined as:

$$J_{\mathrm{TV}}=\frac{1}{2}{\left(\psi -\psi_{e}\right)}^{2}$$ (10)

which penalizes angular deviation between the current heading and the direction toward.

To optimize this objective with respect to the UAV position and heading, we derive the gradient of $J_{\mathrm{TV}}$ with respect to $p_{\mathrm{ego}}\in {\mathbb{R}}^{3}$ and the yaw angle ψ. Let $\text{r }\text{= }{\text{p}}_{c}-p_{\mathrm{ego}}$; then, the gradient with respect to position is given by:

$$\nabla_{p_{\mathrm{ego}}}J_{\mathrm{TV}}={\left[\left(\psi -\psi_{e}\right)\;\frac{-r_{y}}{r_{x}^{2}+r_{y}^{2}},\;\left(\psi -\psi_{e}\right)\;\frac{r_{x}}{r_{x}^{2}+r_{y}^{2}},\;0\right]}^{⊤}$$ (11)

where the zero in the third component reflects that the cost is invariant to motion along the z axis, and the gradient with respect to the yaw angle is:

$$\frac{\partial J_{\mathrm{TV}}}{\partial \psi }=\psi -\psi_{e}$$ (12)

#### Tether clearance

To ensure a safe separation between the UAV and the tether during the binding maneuver, we introduce a clearance cost that penalizes proximity to the tether. We model the tether locally as a 3D line segment defined by the observed tether center $p_{c}\in {\mathbb{R}}^{3}$ and a unit direction vector $d\in {\mathbb{R}}^{3}$. Given the UAV position $p_{\mathrm{ego}}\in {\mathbb{R}}^{3}$, the signed displacement vector orthogonal to the line is computed as:

$$\delta =\left(I-dd^{⊤}\right)\left(p_{\mathrm{ego}}-p_{c}\right)$$ (13)

and the corresponding perpendicular distance is:

$$\text{dist}\left(p_{\mathrm{ego}},\ell \right)=\left\|\delta \right\|$$ (14)

The TC cost is defined as:

$$\begin{array}{c} J_{\mathrm{TC}}\left(p_{\mathrm{ego}}\right)=\frac{1}{2}\cdot {\left(\text{max},\left(0,d_{\mathrm{TC}}-\text{dist}\left(p_{\mathrm{ego}},\ell \right)\right)\right)}^{2} \end{array}$$ (15)

where $d_{\mathrm{TC}}>0$ is a user-specified minimum clearance threshold. The gradient of $J_{\mathrm{TC}}$ with respect to $p_{\mathrm{ego}}$ is given by:

$$\begin{array}{c} \frac{\partial J_{\mathrm{TC}}}{\partial p_{\mathrm{ego}}}=\text{max}\left(0,d_{\mathrm{TC}}-∥\delta ∥\right)\cdot \left(\frac{-\delta }{∥\delta ∥}\right) \end{array}$$ (16)

which smoothly pushes the UAV away from the tether when it gets too close.

### Knotting trajectory optimization

In this work, we adopt the trajectory class $T_{\text{MINCO}}^{s}$ [33] to represent and optimize the trajectory. An s-order MINCO trajectory is a $\left(2s-1\right)$-degree polynomial, which is proven to be the unique optimal solution of the minimum control problem for a chain of s-integrators while being continuously differentiable up to order s−1. Such a trajectory can be compactly parameterized by a set of intermediate points and time durations $\left\{q,T\right\}$. The trajectory generation is then formulated as the following optimization problem:

$$\underset{q,T}{\text{min}}\;J_{s}+\rho T+\;\int_{0}^{T}(J_{D}+J_{K})dt$$ (17)

where $J_{s}$ denotes the smoothness cost directly obtained from the polynomial trajectory, ρ is the time regularization parameter, T is the total trajectory duration, $J_{D}$ represents the cost induced by the UAV’s dynamic feasibility and safety constraints, and $J_{K}$ corresponds to the knotting-specific cost terms. The detailed formulations of each term are as follows:

$$J_{D}=\lambda_{D}\left[J_{c},\;J_{v},\;J_{a},\;J_{\omega }\right]$$ (18)

$$J_{K}=\lambda_{K}\left[J_{\mathrm{EP}},\;J_{\mathrm{TV}},\;J_{\mathrm{TC}}\right]$$ (19)

where $\lambda_{D},\lambda_{K}$ are the predefined weight vectors. $J_{c},J_{v},J_{a},J_{\omega }$ denote the costs for collision avoidance, velocity, acceleration, and angular velocity limits, respectively. $J_{\mathrm{EP}}$ is the EP cost defined in Eq. (7), $J_{\mathrm{TV}}$ is the TV cost defined in Eq. (10), and $J_{\mathrm{TC}}$ is the TC cost defined in Eq. (15). In the object enclosing state, the weights of $J_{\mathrm{TC}}$ and $J_{\mathrm{TV}}$ are set to zero. In the tether binding state, the weight of $J_{\mathrm{EP}}$ is set to zero.

We adopt $T_{\text{MINCO}}^{4}$ to represent the position trajectory $p_{\mathrm{ego}}\left(t\right)={\left[x\left(t\right),\;y\left(t\right),\;z\left(t\right)\right]}^{⊤}$ and $T_{\text{MINCO}}^{2}$ to represent the yaw trajectory $\psi \left(t\right)$, following the formulation in Ref. [33]. The formulations of cost terms not specific to knotting follow [34]. The yaw initialization is obtained by linearly interpolating, over time, between the yaw angles at the initial and target positions.

## Results

The viability of the proposed system was assessed through both simulations and real-world experiments. Its practical applicability was specifically validated by a successful heavy object lifting experiment in a real-world setting. To quantitatively demonstrate the generality of the knot planner, simulations were conducted involving knots of various shapes. Furthermore, ablation analyses were performed to substantiate the design rationale and verify the necessity of key components.

### Lifting a heavy object via autonomous knotting

**Fig. 4.**
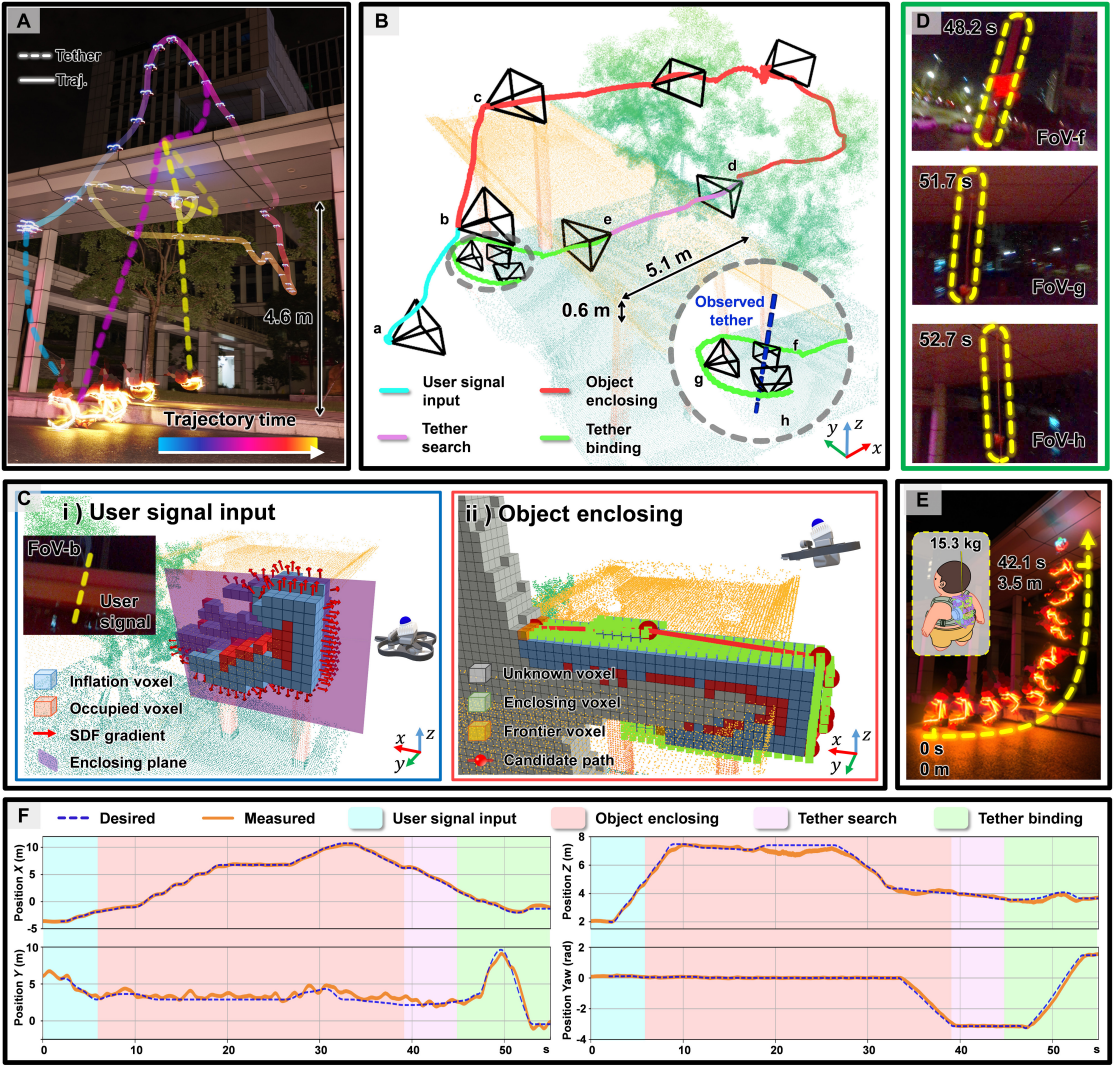
Autonomous knotting and object lifting in an unseen environment. (A) Sequential snapshots of the UAV and object trajectories during knotting and lifting. The solid color-gradient line shows the UAV’s position over time; the dashed line of the same color indicates the corresponding tether state. (B) Detailed knotting process, with trajectory segments colored by task stage: user signal input, object enclosing, tether search, and tether binding. Key UAV poses (a to h) are visualized with FoV. (C) Internal process visualization: (i) extraction of the enclosing plane based on user signal and SDF gradients at position (b); (ii) object enclosing planning with frontier and candidate paths at location (c). (D) Front-view camera images at 3 key moments (f to h) in tether binding, where yellow dashed lines highlight the visible tether in the FoV. (E) Snapshots of the object during lifting, with a 15.3-kg payload raised 3.5 m in 42.1 s. (F) Comparison of desired and measured UAV states. Shaded backgrounds indicate task states. Root mean square error: *X* = 0.174 m, *Y* = 0.109 m, *Z* = 0.229 m, Yaw = 0.121 rad.

The proposed system enables autonomous knotting and heavy-load transport in real-world unseen environments. As depicted in Fig. 4A, the experiment was conducted within an urbanized environment featuring buildings, a covered linkway, and trees. The knotting target was the roof of the linkway whose cross section measured 5.1 m in width and 0.6 m in height, supported by some pillars at the height of 4.6 m. The transported payload simulated a “maintenance technician”, represented by a 15.3-kg weighted safety-harnessed cartoon figurine. All UAV-related modules, including perception, state estimation, mapping, tether detection, and trajectory planning, were executed in real time on the onboard computer (Nvidia Orin NX). For the planner, we employed the L-BFGS algorithm to solve the trajectory optimization problem.

Fig. 4B illustrates the flight trajectories and corresponding attitude changes across different states. By interpreting the human-given command illustrated in Fig. 4C-i, the system first projected the command into the world coordinate system and determined the enclosing plane based on the SDF of the observed knotting target. Then, the system entered the object enclosing state, where it iteratively selected candidate viewpoints to eliminate surface frontiers near the enclosing plane as shown in Fig. 4C-ii and completed the enclosing process. Once all surface frontiers around the enclosing plane were eliminated, the system transitioned to the tether search state, during which it returned toward the initial position while attempting to detect the tether. To enable robust detection, visual markers were affixed to the tether, and their locations were identified using YOLOv11 [35]. These detections were then fused with depth image data to estimate the state of the tether. Upon successful localization, the system proceeded to the tether binding state.

Leveraging the designed TV and TC metrics (Knotting trajectory generation section), the system safely completed the bind operation. Throughout this process, as shown in Fig. 4D, the tether and markers remained clearly visible for most of the operation. This visibility ensured that real-time adjustments to the binding path could be made based on the tether’s configuration, and this will be further discussed in Results. Subsequently, the winch tightened the tether, lifting the 15.3-kg load 3.5 m off the ground within 42.1 s as shown in Fig. 4E.

To mitigate the risk of aggressive tether motion induced by rapid UAV motion, the system generates trajectories that are as smooth and gradual as possible. Fig. 4F illustrates the measured UAV pose alongside the planned trajectory, with different operational states indicated using color-coded ribbons. Despite deviations caused by control limitations and tether-induced disturbances, the system successfully completed the knotting task. These results underscore the robustness of the proposed algorithm and demonstrate its practical applicability in the real-world environment.

### Knotting on geometrically diverse structures

**Fig. 5.**
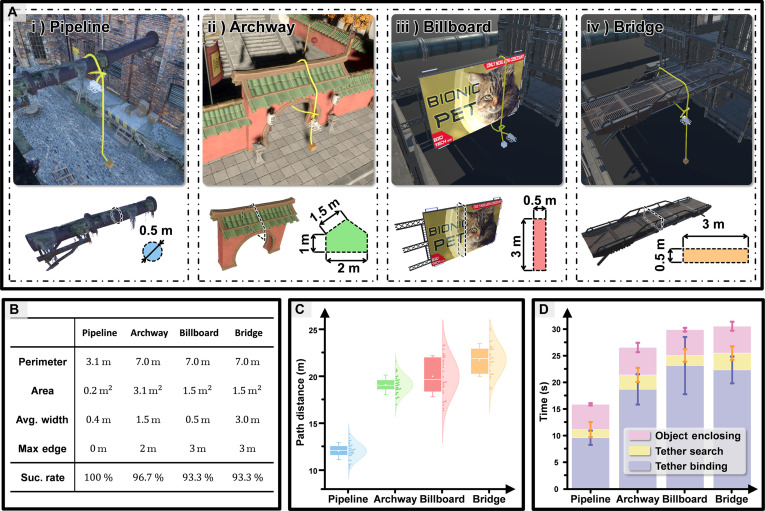
Various structural target knotting experiments. (A) Knotting results on 4 distinct structural targets. (B) Statistics describing the geometrical feature of knotting target and the success rate in 30 trials. (C) Comparison of knotting path lengths across different targets. (D) Temporal profiles of operational states.

The previous experiment shows the field-tested reliability of the proposed system. In comparison, the experiment presented in Fig. 5 aims to validate the system’s geometry-agnostic knotting capability. As shown in Fig. 5A, the system successfully performed knotting operations on 4 structurally distinct targets, commonly used in real world, including a pipeline, archway, billboard, and bridge, demonstrating its ability to generalize across diverse geometries. To assess the consistency and reliability of this capability, 30 trials were conducted for each target configuration (Fig. 5B). The system achieved success rates exceeding 90% across all scenarios, confirming the robustness and repeatability of its knotting performance under varying shapes.

The path distance (shown in Fig. 5C) demonstrates that smaller cross-section targets (e.g., pipelines) require shorter knotting paths. In contrast, for structures with equivalent cross-sectional perimeter, extended planar surfaces with long edges, such as billboard and bridge, exhibited significantly longer paths than those with shorter angled facets (e.g., archway). This divergence arised directly from FoV constraints and limited sampling rates, as these limitations necessitated repetitive back-and-forth motions to re-observe unknown regions. Consequently, extended planar structures require both more intensive zigzag scanning and more frequent corrective motion during the object enclosing state to fully explore the enclosing plane. These compounded effects collectively increased knotting path lengths and amplified metric variation, as further evidenced by the duration data across operational states.

The average temporal profiles of operational states across scenarios (Fig. 5D) identify the object enclosing state as the primary contributor to temporal disparities, with its duration closely aligned with the traveling distance trends: Extended planar structures consistently require prolonged enclosing phases and longer knotting paths. In contrast, the tether search state and tether binding state exhibited consistently relatively low duration variability across all scenarios. This geometry-insensitive behavior further corroborates the system’s shape-agnostic knotting capability.

### Ablation studies

This subsection provides a comprehensive overview of the core algorithm’s functionality, including the EP metric and binding-related components (specifically TV and TC metrics). The necessity of these features was validated through a series of ablation experiments.

#### Enclosing planarity

**Fig. 6.**
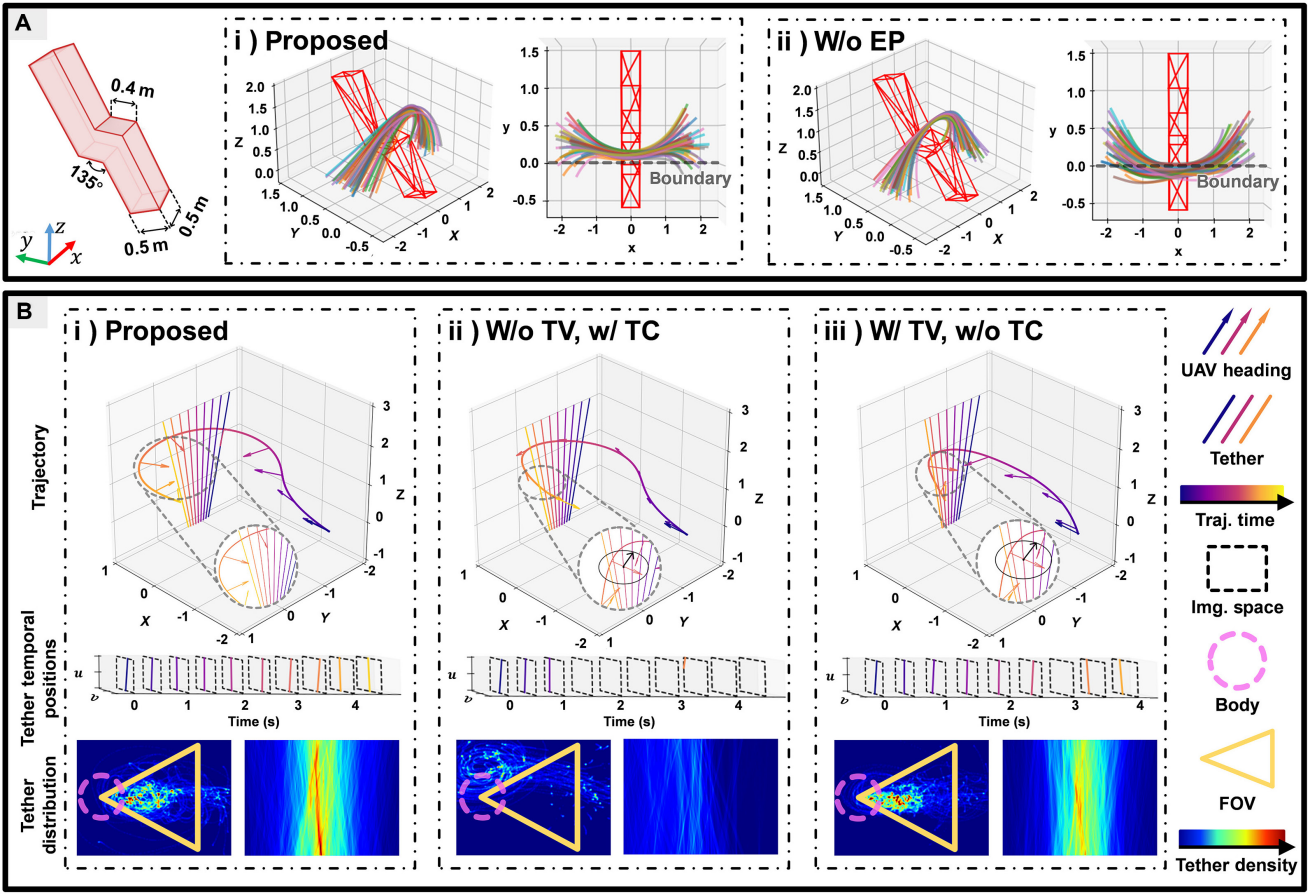
Ablation analysis. (A) Comparison of knotting trajectories with and without EP. The left panel shows the compact folding binding geometry used as the knotting target. Subfigures (i) and (ii) display trajectory distributions from 50 trials. Multicolored curves represent individual UAV trajectories, with the red envelope indicating the target geometry. Dashed lines in the top view mark the tether slippage boundary of the central transition platform. (B) Binding performance across 3 experimental configurations: (i) proposed (w/ TV, w/ TC), (ii) TC-only (w/o TV, w/ TC), and (iii) TV-only (w/ TV, w/o TC). Trajectories are time-encoded by color, with arrows showing UAV heading and slanted lines indicating tether. Aggregated tether distributions over 50 trials are shown in the UAV body frame, with the dashed circle denoting the UAV body and the yellow triangle indicating the FoV. These visualizations reveal both tether-body collisions and detections. The tether distribution in FoV further illustrates the effect of binding metric on TV and perception.

Ablation simulation experiments were conducted to evaluate the functional role of EP in the constrained environment. As shown in Fig. 6A, the knotting target was a monolithic bent prismatic structure with a continuous spatial folding angle of 135°. A horizontal transition platformed at its center, measuring 0.4 m × 0.5 m, introduced a spatial offset between the 2 arms of the structure.

The UAV was tasked with performing a knotting motion across the cross-sectional plane of the transition platform, as illustrated in Fig. 6A(i and ii). During the operation, the trajectory must remain strictly within the spatial boundary of the platform; deviations beyond this region may cause the tether to lose structural support and slip off the geometry, leading to knotting failure. The UAV’s initial position was randomly sampled from a circular disk of radius 0.5m centered at $\left(x=-1.7\;m,\;y=0.3\;m,\;z=0.2\;m\right)$. The target position was similarly sampled from a symmetric disk of the same radius, centered at $\left(x=1.7\;m,\;y=0.3\;m,\;z=0.2\;m\right)$.

The performance of the proposed method with EP was compared with a baseline lacking this feature. As shown in Fig 6A(i and ii), in 50 trials, trajectories generated using EP were more compact and remained better constrained within the boundary, whereas those generated without EP exhibited notable dispersion and frequent violations of the spatial limits. Specifically, with EP, the UAV succeeded in all 50 trials (100% success rate), while without EP it succeeded in only 4 trials (8% success rate). These results demonstrate that EP significantly improves trajectory compactness and boundary adherence, both of which are critical for ensuring successful knotting in constrained environments. These results demonstrate that EP significantly improves trajectory compactness and boundary adherence, both of which are critical for ensuring successful knotting in constrained environments.

#### Tether visibility and tether clearance

TV is designed to keep the tether within the UAV’s FoV, enabling real-time position monitoring and dynamic path adjustments essential for successful binding under tether movement. In parallel, TC provides a safety buffer by maintaining minimum distance thresholds to mitigate collision risks.

An additional ablation experiment was conducted to evaluate the necessity of TV and TC during the binding process, as illustrated in Fig. 6B. Under consistent initial conditions, UAVs performed binding tasks on kinematically stochastic tethers across 3 experimental configurations: (a) proposed (w/ TV and TC); (b) TC-only (w/o TV); and (c) TV-only (w/o TC). Across 50 binding trials, the proposed system achieved 45 successful bindings, compared to 35 and 23 for the TV-only and TC-only groups, respectively. This performance gap highlights the importance of both TV and TC for robust binding.

In the TC-only condition, although the collision was less than in the TV-only group, the system exhibited deficiencies in tether perception. Analysis of tether temporal positions and heatmap distributions revealed that visual contact with the tether was only sporadically maintained. This forced the system to rely on memorized positional data during perception loss, resulting in 2 primary failure modes, as shown in the trajectory: (a) binding attempts outside the predefined topological boundaries due to tether movement; and (b) collisions caused by delayed response during tether motion. These failure modes underscore the necessity of TV for reliable tether state estimation.

In the TV-only configuration, the perception performance of the tether was comparable to that of the proposed system, with visual contact reliably maintained for continuous estimation. However, the absence of TC led the UAV to operate dangerously close to the tether, frequently resulting in entanglement failures when interacting with tether motion. The proposed system overcomes these limitations through coordinated functionality: TV provides persistent spatiotemporal tether tracking, while TC ensures spatial safety. Together, they enable robust binding performance in stochastic environments.

## Discussion

In this work, we proposed a transport system that integrates a tethered UAV and a winch, enabling object transport by autonomously knotting the tether around surrounding environmental structures and subsequently using the winch to reel it in. We developed a fully autonomous knotting method that includes a human-in-the-loop enclosing strategy and a robust binding module. Real-world experiments demonstrated the system’s feasibility, while extensive simulations validated its geometry-agnostic performance across diverse targets.

The proposed EP metric was shown to significantly improve the compactness and spatial controllability of the knotting trajectories, effectively reducing the likelihood of tether slipping outside the target enclosing region and ensuring successful engagement. In the tether binding state, the integration of TC and TV proved critical: TV supports persistent spatiotemporal tracking of the tether, while TC guarantees a safety buffer that prevents entanglement and collision, together enhancing binding robustness under dynamic uncertainties.

Despite these promising results, several limitations remain. The current system assumes reliable low-level flight control and relatively stable environmental conditions. The perception pipeline also relies on clear visual access to the tether, which may degrade in cluttered or high-speed scenarios. Additionally, the winch–tether mechanism may require further mechanical optimization to improve reeling efficiency and tension control during high-load transport.

To enable full deployment in unstructured environments, future work should integrate advanced control algorithms capable of compensating for the disturbances introduced by the tether. In addition, trajectory planning should more explicitly account for the dynamic interactions between the tether and the surrounding environment, especially in cluttered or contact-rich settings. Furthermore, improving the accuracy and robustness of thin tether detection and pose estimation remains a critical challenge, particularly under adverse visual conditions such as occlusions, motion blur, or limited illumination. Future efforts may explore multimodal sensing strategies to enhance perception reliability.

## Data Availability

The data used to support the findings of this study are available from the author upon reasonable request.

## Supplementary Materials

Supplementary 1 Supplementary Text Figs. S1 to S3 Table S1 References [38–40]
