# Supplementary material for: Tethered UAV Autonomous Knotting on Environmental Structures for Transport
Source: Cyborg Bionic Syst. 2025 Dec 26;6:0450. doi: 10.34133/cbsystems.0450 (PMC12741257; doi:10.34133/cbsystems.0450)
Supplement: Supplementary 1 — Supplementary Text Figs. S1 to S3 Table S1 References [38–40] [file cbsystems.0450.f1.docx]

### Derivations of Radar Chart Metrics

Table S1: Key specifications of different UAV-related platforms. The maximum flight time refers to the duration under maximum payload. For flight time and battery capacity, the data of Blowfish A2G are provided by the distributor and the user manual, while for DJI FC30, the reported values correspond to the single-battery mode under extreme conditions.

| **Parameter** | **DJI FC30**[38] | **Cubix**[13] | **Blowfish A2G**[39] | **Proposed** |
| --- | --- | --- | --- | --- |
| Self-weight (kg) | 53.75 | 7.66 | 22 | 3.5 |
| Max Payload (kg) | 40 | 139.24 | 12 | 245 |
| Max Flight Time | 8 min | N/A | 28 min | N/A |
| Battery Capacity | 1984.4 Wh | – | 1965.6 Wh | – |

Step-by-step derivations of the metrics illustrated in the radar chart in Fig. 1, using the platform specifications summarized in Table 1, are provided below.

**DJI FC30 (single battery mode):** Carrying 40 kg for 8 min results in efficiency

$$\eta=\frac{1984.4 \text{Wh}}{8 \text{min}\times40 \text{kg}}=372.08 \text{W/kg}.$$

Given self-weight of 53.75 kg, the payload-to-weight ratio is

$$\text{PWR}=\frac{40}{53.75}=0.744.$$

**Cubix:** Each wire module can sustain a continuous tension of $180 \text{N}$. With 8 modules, the theoretical maximum payload is

$$\text{MaxPayload}=\frac{8\times180}{9.8}-7.66\approx139.3 \text{kg}.$$

Given the self-weight of $7.66 \text{kg}$, the payload-to-weight ratio is

$$\text{PWR}=\frac{139.3}{7.66}\approx18.19.$$

The required motor current can be derived from the relationship between the desired wire tension and the actuator parameters. According to the equations in Cubix [13],

$i=\frac{r}{\mu_{p}\mu_{G}GK_{t}} f_{\text{ref}}^{\text{final}},$ (20)

where *r* is the winch radius, $\mu_{p}$ and $\mu_{G}$ are the pulley and gear efficiencies, *G* is the gear reduction ratio, $K_{t}$ is the motor torque constant, and $f_{\text{ref}}^{\text{final}}$ is the required wire tension.

For CubiX, the parameters are given as

$$r=0.008 \text{m}, G=53, K_{t}=0.014 \text{N/A}, f_{\text{ref}}^{\text{final}}=180 \text{N}.$$

The required motor current under maximum continuous tension can be directly computed as

$i=\frac{0.008\times180}{1.0\times1.0\times53\times0.014}\approx1.94 \text{A}.$ (21)

With a supply voltage of $U=48 \text{V}$, the efficiency is obtained as

$\eta=\frac{P}{m}=\frac{UI}{m}=\frac{48\times1.94}{\frac{180}{9.8}}\approx5.07 \text{W/kg}.$ (22)

**Blowfish A2G (single battery mode):** Carrying 12 kg for 28 min results in efficiency

$$\eta=\frac{1965.6 \text{Wh}}{28 \text{min}\times12 \text{kg}}=351.00 \text{W/kg}.$$

Given self-weight of 22 kg, the payload-to-weight ratio is

$$\text{PWR}=\frac{12}{22}=0.545.$$

**Proposed Platform:** Based on the operating curve of the GIM8108-36 motor [40], we select the operating point of $90 \text{N}$ $m$ torque at $40 \text{rpm}$, corresponding to an output power of $380 \text{W}$. Given the winch radius of $3.75 \text{cm}$, the maximum payload can be calculated as

$$m=\frac{90}{0.0375\times9.8}-3.5\approx241.4\text{kg},$$

where $3.5 \text{kg}$ is the self-weight of the platform. So the payload-to-weight ratio is:

$$\text{PWR}=\frac{241.4}{3.5}\approx68.97.$$

Accordingly, the load efficiency is expressed as

$$\eta=\frac{380}{241.4}\approx1.57\text{W/kg}.$$

These derivations clarify the assumptions and make explicit whether values come from theoretical upper bounds or empirical endurance/load tests.


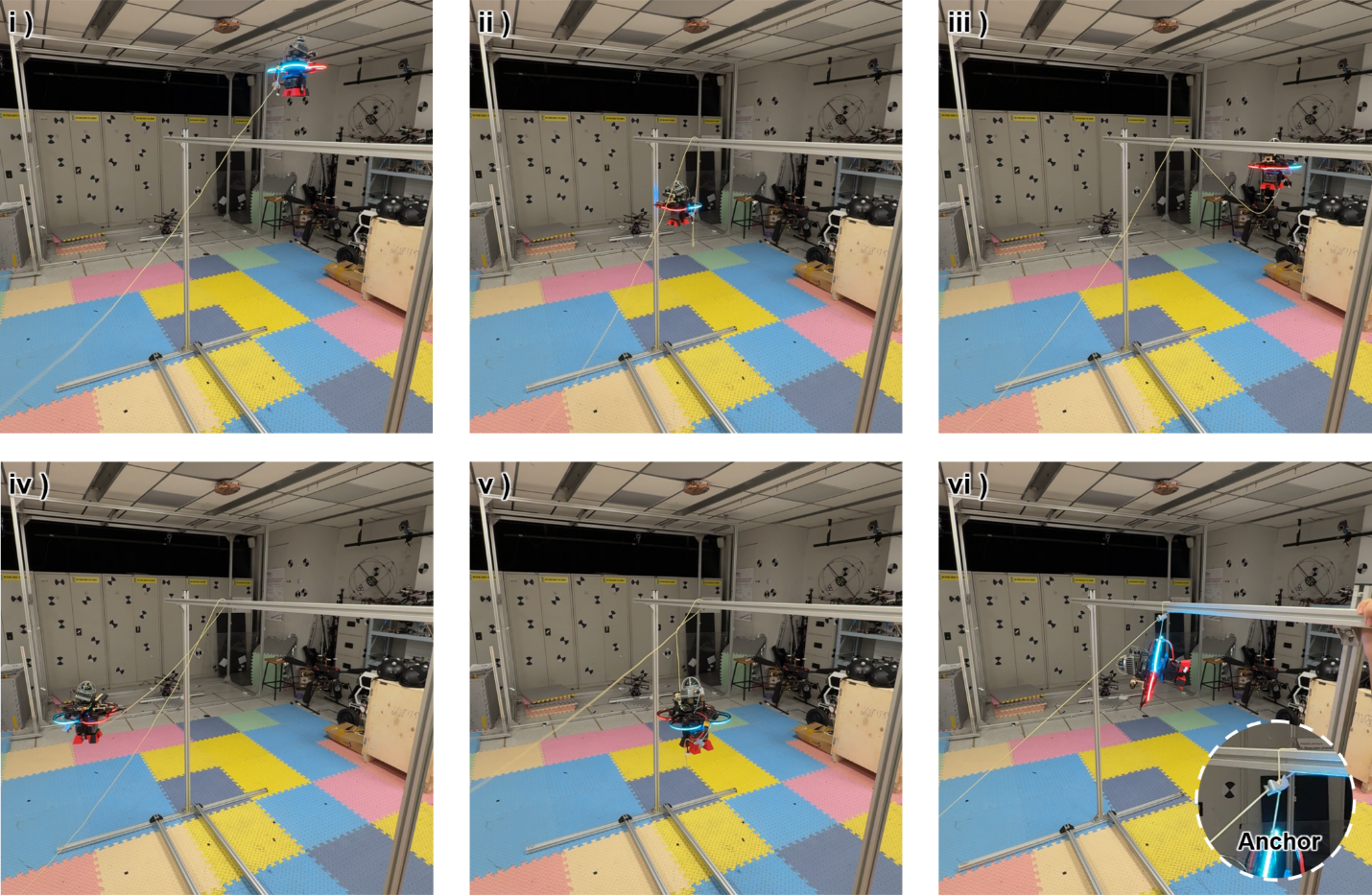


Figure S1: Knotting process. The UAV first guides the tether to encircle the knotting target once, then loops around the tether itself and suspends to create a crossing, and finally pulls the tether tight under tension so that an anchor is formed and fixed in place.

### Knotting Process

In this work, we follow the same method described in [13]. Specifically, the UAV first circles once around the target object to wrap the tether, after which it suspends itself to create a crossing of the tether. Finally, the UAV applies tension by leveraging its own gravity together with the onboard winch, thereby tightening the tether so that the anchor achieves entanglement and completes the knotting. The knotting process is illustrated in Fig. 7.

### Tether Detection

We use the depth and RGB images from an RGB-D camera (Realsense D435i) together with YOLOv11 [37] to detect the tether. The experimental results of tether detection are shown in Fig. 8.


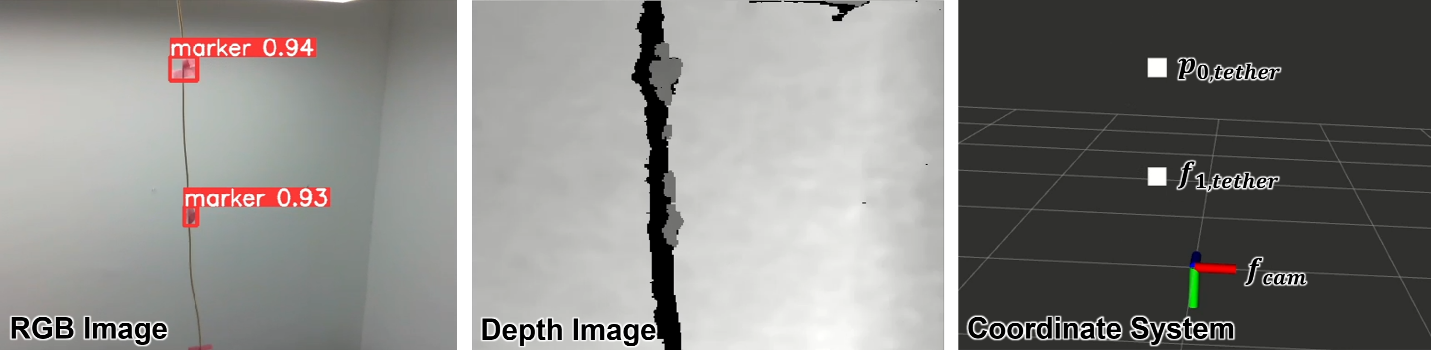


Figure S2: Tether detection results.

### Figure-Eight Trajectory Tracking

Fig. 9 shows the result of the figure-eight trajectory tracking experiment conducted to validate the UAV’s control performance in real-world conditions. The trajectory has a size of $3.5\times3.0$ m, and the controller parameter settings were kept identical to those used in the real-world experiments. The UAV achieved stable tracking with an average position error (APE) of *0.0713* m.


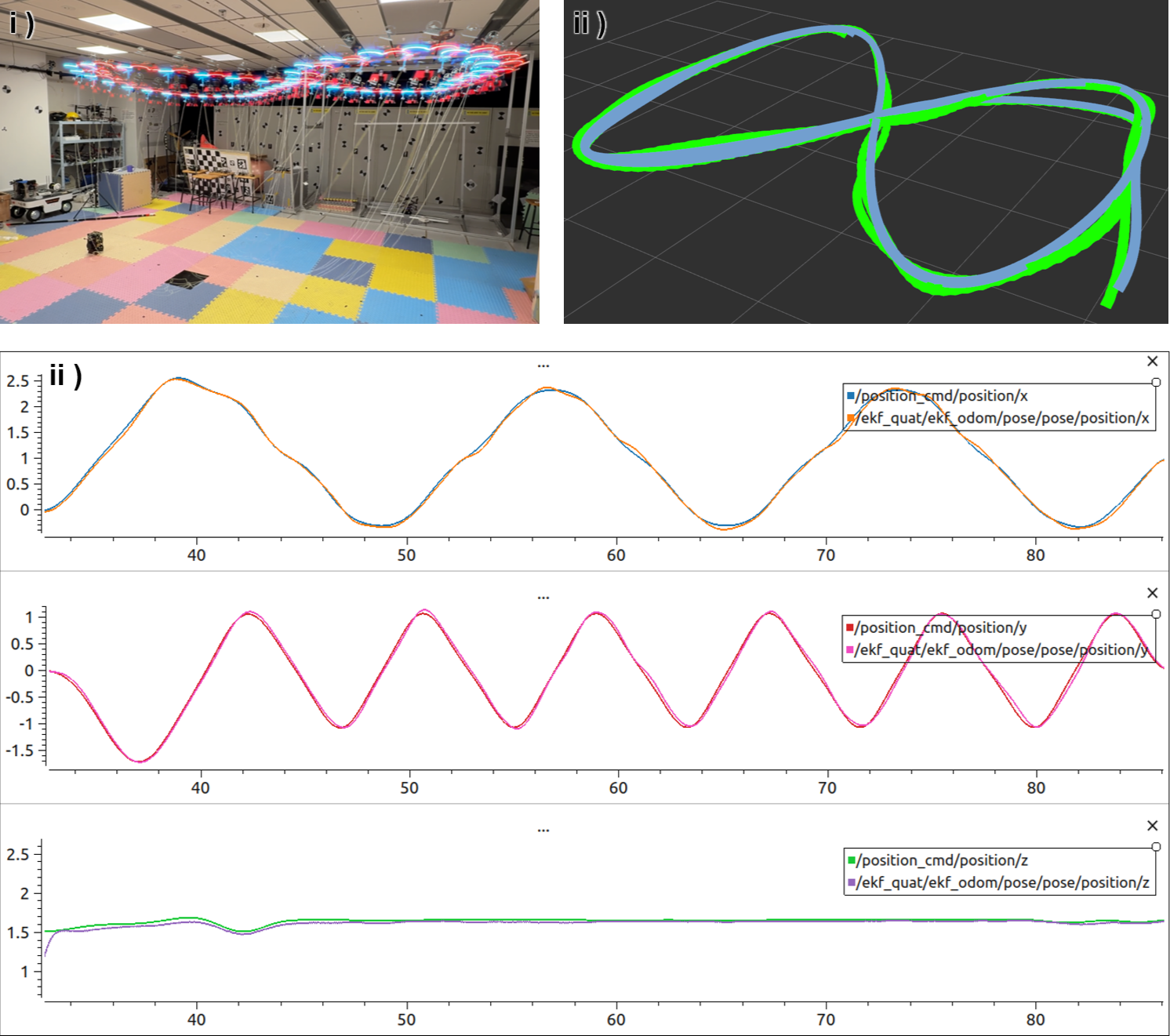


Figure S3: Figure-eight trajectory tracking experiment. (i) The UAV, carrying a tether, follows a figure-eight trajectory using radar-based localization. (ii) Visualization of the results, where the blue curve denotes the desired trajectory and the green curve indicates the measured trajectory. (iii) Position tracking results in the *x*, *y*, and *z* axes.
